# Conceptualisation of restrictive repetitive behaviours across autism and orthorexia nervosa

**DOI:** 10.3389/fpsyt.2026.1936477

**Published:** 2026-10-28

**Authors:** Jane Edge, Jac Airdrie, Maddy Greville-Harris, Samuel J.R.A. Chawner

**Affiliations:** 1 Wolfson Centre for Young People’s Mental Health, Department of Psychological Medicine and Clinical Neuroscience, Cardiff University, Cardiff, United Kingdom; 2 School of Psychology, Cardiff University, Cardiff, United Kingdom; 3 Psychology Department, University of Exeter, Washington Singer Building, Exeter, United Kingdom; 4 Centre for Neuropsychiatric Genetics and Genomics, Department of Psychological Medicine and Clinical Neurosciences, Cardiff University, Cardiff, United Kingdom

**Keywords:** autism, cognitive inflexibility, conceptualisation, intolerance for uncertainty, orthorexia nervosa (ON), perfectionism, restrictive repetitive behaviours (RRBs)

## Abstract

Orthorexia Nervosa (ON) is widely regarded as an emerging eating disorder although it is not currently recognised as a formal diagnosis. It is characterised by a strict adherence to “pure” eating which becomes excessive and can lead to nutritional deficiencies, psychological distress, and impaired daily functioning. Restrictive eating behaviour in ON shows overlap with Restricted Repetitive Behaviours (RRBs) observed in autism. However, the conceptual relationship between ON and autism remains underexplored. This perspective article synthesises existing literature to explore potential overlapping and diverging mechanisms of RRBs across ON and autism. Intolerance for Uncertainty is highlighted as a possible shared mechanism that may contribute to RRBs observed in both ON and autism, whereas Cognitive Inflexibility and Perfectionism may mechanistically differentiate the conditions. We consider that autistic RRBs reflect neurodevelopmental divergence, whereas orthorexic RRBs may be influenced by evaluative belief-centred cognitions. We propose a conceptual model outlining how cognitive, affective and functional factors may interact to produce and maintain RRBs. Clarifying these distinctions is important in supporting clinicians to appropriately consider the drivers and mechanisms underlying RRBs across ON and autism, therefore supporting accurate diagnosis, formulation and treatment.

## 1 Introduction

Orthorexia Nervosa (ON) is widely considered an emerging eating disorder, characterised by an obsessive focus on health and strict adherence to “pure” eating (1). Although not formally recognised in DSM (2) and ICD (3) classification systems, orthorexic tendencies are associated with negative outcomes including malnutrition, social impairment, and poorer quality of life (4). Qualitative research suggests patterns of repetition and restriction are characteristic of the presentation of ON with those experiencing ON reporting, “*I’ll stick to the same thing all the time*” and “*I don’t try new things*.” (5). Individuals with ON describe compulsive checking of ingredients, extreme adherence to healthy eating and distress when “safe foods” are unavailable (1). In turn, this can result in weight loss and body image difficulties, with individuals reporting, “I got really skinny ‘cause I was restricting myself so much” (6). Such restrictive behavioural traits exhibit similarity with Restrictive Repetitive Behaviours (RRBs) seen in autism. However, research into the overlap of ON and autism is limited, despite a growing body of literature investigating the overlap between eating disorders and autism (7). Autism is a neurodevelopmental condition categorised by differences in thinking, communication, and behaviour (8). RRBs are a core feature of autism, supporting sensory regulation, the expression of specific interests, and maintenance of predictability (9). Whilst RRBs are a diagnostic feature of autism, they are marked by considerable heterogeneity, whereby the specific behaviours expressed, and the intensity of behaviours, vary across the autistic community (10).

While this paper focuses on RRBs broadly as a behavioural aspect of autism, prior work indicates that RRBs in autism, such as rigid routines surrounding mealtimes, could lead to restrictive eating disorders (11). Furthermore, research consistently finds that autistic individuals are at substantial increased risk of developing eating disorders, with recent findings indicating that 23% of people with an Anorexia Nervosa (AN) diagnosis are also autistic (12). One explanation for this over-representation of autistic individuals in eating disorder populations focuses on RRBs, proposing that the need for routine, specific rules surrounding food, and sensory sensitivities all exacerbate eating disorder risk (11). Despite heightened risk of eating disorders for autistic people, and behavioural parallels between RRBs in autism and ON, the conceptual formulation between the two conditions is yet to be thoroughly examined. The aim of this perspective is to synthesise the current literature and outline potential conceptualisations of the relationship between RRBs, autism, and ON.

## 2 The classification of orthorexia nervosa

Before conceptualising the overlap between ON and autism it is important to recognise the ongoing debate regarding diagnostic classification for ON. Central to this debate is the assumption that overlapping characteristics indicate ON is not a distinct condition, but a manifestation of a pre-existing condition. For example, some draw parallels between orthorexic tendencies and obsessive preoccupations, proposing ON as a subtype of obsessive-compulsive disorder (OCD) (13). However, a systematic review investigating the magnitude of associations between orthorexic tendencies with disordered eating and OCD, posits ON symptoms are more strongly associated with eating disorders than with OCD (14).

Although ON is yet to be formally recognised in DSM and ICD classification systems, initial evidence suggests heterogeneity with individuals exhibiting orthorexic tendencies differing in motivations, eating behaviour, and levels of impairment (5, 15). As such, assessment tools used to capture ON vary (15), with preliminary evidence highlighting mixed results regarding validity (16–18).

However, justification for treating ON as a separate eating condition is supported by clinicians’ experiences. High levels of consensus amongst international clinicians on the diagnostic criteria for ON suggests ON can be defined by a distinct set of diagnostic criteria, representing a unique clinical presentation (19). Further research points to consistency in proposed diagnostic criteria for ON across studies. This criteria includes; “(a) obsessional or pathological preoccupation with healthy nutrition (b) emotional consequences of non-adherence to self-imposed nutritional rules and (c) psychosocial impairments in relevant areas of life, malnutrition, and weight loss (4).

Although we acknowledge conceptualising ON without formal diagnostic classification is complex, due to growing evidence for the condition and consistency in proposed diagnostic criteria, we will consider ON as a potential distinct eating disorder. However, we will draw on other eating disorder research in areas where ON specific research does not yet exist.

## 3 Rationale

The relationship between autism and disordered eating behaviour is multifaceted, interacting in complex ways and varying between individuals (19, 20). However, patterns of routines, rule following and restriction highlights RRBs as a specific point of behavioural overlap across conditions. It is therefore necessary to examine whether these similarities arise from similarly shared mechanisms or from developmentally divergent pathways. Furthermore, discussion of RRBs across conditions enables evaluation of their functionality. It is important to recognise that RRBs are not innately adaptive nor maladaptive in either condition. Rather, exploration of the contexts in which they occur, alongside their impact on functioning, is required to understand RRBs in both autism and ON.

We do not assume that RRBs across ON and autism are equivalent, but aim to delineate where RRBs mechanistically overlap and diverge, focusing on Intolerance of Uncertainty (IU) (21, 22), Cognitive Inflexibility (23, 24) and Perfectionism (25, 26) as underlying drivers of RRBs. This will frame a transdiagnostic discussion surrounding the differing functions and reasons why RRBs arise and are maintained across conditions. Clinical implications will be discussed, arguing that advancing understanding of RRBs will aid in informing assessment, diagnosis, clinical formulation and development of ON treatment.

## 4 Mechanisms of restricted repetitive behaviours

RRBs in autism are underpinned by a diverse interplay of mechanisms, spanning cognitive, affective and neurobiological domains (9). As for orthorexic tendencies, research into mechanisms is very limited. However, initial research indicates that similarly rigid cognitive styles may be contributing to the patterns of restriction seen in ON (5). This section will explore IU as a potential area in which autism and ON mechanisms of RRBs show the greatest similarity, before outlining cognitive inflexibility and perfectionism as mechanisms that potentially differentiate autistic and orthorexic RRBs.

### 4.1 Intolerance for uncertainty and anxiety

IU is a cognitive bias whereby unfamiliar or ambiguous stimuli, such as new environments, evoke distress (27). IU often presents through checking and re-checking, overplanning, and avoidance of new situations (21). These behaviours are maintained by patterns of rumination and catastrophising, resulting in strong associations with anxiety (28). While often discussed within the context of generalised anxiety disorder, IU is regarded as a transdiagnostic feature exhibited in many conditions, including eating disorders (29) and autism (30).

In autism, IU is a core cognitive mechanism believed to be underpinning RRBs, linked to the “insistence of sameness” subtype of RRBs due to shared difficulty surrounding the unfamiliar and desire to maintain predictability. A systematic review of the relationships between RRBs, IU and anxiety in autism found significant positive correlations between IU and RRBs, as well as between IU and anxiety, and RRBs and anxiety (31). This aligns with South and Rodgers model (32) which positions IU as a mediator for “*all sensory, emotional and cognitive factors predicting autistic anxiety*.” The model indicates a unidirectional pathway from IU to RRBs, reinforcing IU as a mechanistic driver. It additionally describes a bidirectional relationship between RRBs and anxiety, whereby rising anxiety increases RRBs, and being unable to perform RRBs, in turn, heightens anxiety.

Although research into IU in ON is limited, qualitative research suggests that RRBs in ON reflect an anxiety-driven response to uncertainty and a broader strategy for managing intense emotional experiences (33, 34). Individuals with orthorexic tendencies describe reliance on sameness, “*I don’t try new things either, so I’ll stick to the same thing all the time*” (5). Further qualitative research has explored distress responses when restrictive dietary behaviours are violated, “*I would freak out, likely to be disgusted with myself and feel strong anxiety about the effects*” (35). This need for familiarity in ON, and accompanying negative affect when breached, parallels the experiences of IU for autistic individuals, positioning anxiety management as a shared function of RRBs across conditions. Giles., et al. (22) further identified strong associations between IU, autistic traits and orthorexic tendencies, with mediation analyses indicating IU acts as a key pathway linking autistic traits to orthorexic tendencies. This supports IU as a central transdiagnostic mechanism underpinning RRBs across conditions.

### 4.2 Cognitive inflexibility

Cognitive inflexibility refers to rigid thinking patterns, often presented as difficulty adapting to change and updating beliefs in accordance with new information (36). Although present in both autism and ON, they may differ in their underlying mechanisms.

In autism, cognitive inflexibility represents trait-level neurodevelopmental differences in information processing. This encompasses a wide range of cognitive processes, including differences in set-shifting (37), executive functioning (38) and predictive processing (39). We will focus specifically on Monotropism, not as an exhaustive explanation of autistic cognition underpinning RRBs, but rather as one theoretical perspective of autistic cognition which holds a closer conceptual alignment with RRBs. Monotropism describes the intense narrowing of attention to one or few items (40), describes the intense narrowing of attention to one or few items. Associated with the “fixed interests abnormal in intensity” DSM-5 RRBs criterion, monotropism produces “attentional tunnels” described as “flow state” or “hyper-focus” (41). This can pose difficulty for autistic individuals, especially in new environments, though qualitative research also highlights monotropic attention as positive and joyful for autistic individuals (41).

Autism and ON both show intense attentional fixations on specific areas of interest. However, for ON this cognitive inflexibility is perhaps not equivalent to autistic monotropism. Autistic attentional narrowing varies widely by individual interest, whereas for ON, rigid cognitive patterns surround the specific focus of healthy eating. Although specific beliefs surrounding diet will vary, the overarching attentional bias to pure eating is a core aspect of ON presentation. Despite differences in the nature of the attentional focus, similar patterns of cognitive inflexibility present in restrictive eating behaviour.

Research identifies cognitive inflexibility as a transdiagnostic trait across disordered eating, strongly associated with eating disorder severity (24). Therefore, cognitive inflexibility may also underpin chronic restriction exhibited in ON. Research into cognitive inflexibility in ON is scarce, however looking to the anorexia nervosa (AN) literature for insights, individuals with AN self-reported high cognitive inflexibility yet performed comparatively to healthy controls on cognitive flexibility tests (23). Although this needs testing, this evidence from AN presents an alternative perspective that cognitive inflexibility in ON could arise from subjective beliefs which consequently impact behaviour. Furthermore, where inflexible cognitions in AN surround weight and shape, cognitive inflexibility in ON emerges from beliefs regarding food purity. Therefore, although the lived experience of inflexibility may be shared across conditions, the underlying cognitions driving inflexibility in ON differentiate it from those seen in AN and in autism.

### 4.3 The role of perfectionism

The role of perfectionism is a mechanism potentially more central to the emergence of RRBs in ON compared to autism. We consider perfectionism as a “personality trait” whereby extreme importance is placed on achieving and maintaining unrealistically high standards. Perfectionism operates as a similarly inflexible thinking style, characterised by “all or nothing” ways of thinking, and results in severe emotional distress when these “perfect standards” are not obtained (25, 26).

Perfectionism in ON is experienced as a desire for the “perfect diet” (42), however, the specifics of what makes a certain diet “perfect” and the subsequent “eating rules” vary and are shaped by individual and interpersonal experiences (5). An example from qualitative research is; “*I transitioned from vegetarian to vegan … After that, I went gluten-free to further “perfect” my diet.”* (43) *and “I’m trying to grip on more by applying more rules”* (44).

Novara., et al. (2023) (45) investigated perfectionism as a multi-dimensional personality trait, encompassing perfectionistic strivings; having high personal standards and active pursuit of perfection and perfectionistic concern; rumination over past errors and negative affective responses to imperfection. This found that greater orthorexic tendencies were strongly associated with higher perfectionism across both dimensions (perfectionistic strivings and concern). More recent findings further refine the role of perfectionism, finding the dimension of perfectionistic strivings as a stronger predictor of orthorexic tendencies (42). Crucially, perfectionism does not interact with diet (45) suggesting perfectionism precedes orthorexic cognition, rather than arising from “diet culture” or social environments.

Research investigating perfectionism related concepts in autism, such as fear of failure and rigid standards is growing (46–48). However, studies explicitly addressing perfectionism in relation to RRBs are limited, with research largely focusing on perfectionism as a mediator for co-morbid psychiatric conditions (46, 49). Of research addressing autism and eating disorders, perfectionism is either positioned as unrelated to RRBs or does not address perfectionism as a possible component (50, 51). This could represent a crucial distinction between autism and ON, as perfectionism may be explained as a defining antecedent to RRBs in ON, highlighting a mechanistic divergence between the two conditions. Further investigation into the role of perfectionism and related concepts in the emergence of RRBs in autism is necessary to further clarify this as a possible point of mechanistic distinction between autistic and ON RRBs.

## 5 Integrating mechanisms - proposed pathways

### 5.1 Functions of RRBs

RRBs across autism and orthorexia result from a combination of similar and diverging mechanisms. However, what is of potentially greater importance for clinical formulation is the ways in which autistic and orthorexic RRBs differ by function. This section will discuss two possible explanations as to why RRBs surface and are maintained across the conditions, specifically addressing the role of self-regulation compared to self-evaluation as functions that differentiate RRBs across the conditions.

### 5.2 Self-regulation: a proposed autism pathway

One functional aspect distinguishing autistic RRBs from orthorexic behaviours relates to self-regulation; the ability to manage thoughts, behaviours and emotions (52). In autism, RRBs serve a self-regulatory function, working as an adaptive coping strategy to hypo- or hyperreactivity to environmental stimuli, influenced by differences in sensory processing (53, 54). Research investigating autistic individuals’ experiences of RRBs identified self-regulation of attention, emotion and sensory experience as a core function of RRBs (55). Participants in this study explained that RRBs can act as “*mini distractions … coping mechanisms just to deal with what’s happening*.” Similar patterns of utilising RRBs to self-regulate appear in children with autism, with teachers reporting, “*If something they felt uncomfortable with … or was making them a bit anxious or distressed……they would be doing the RRB … that would be soothing them*.” This positions RRBs in autism as adaptive strategies to cope with sensory input in day-to-day life. Conversely, current research suggests sensory regulation is not a motivator for RRBs in ON, with a recent study finding a negative association between sensorial appeal and orthorexic tendencies (56). However, research regarding possible sensory processing differences in ON is yet to be thoroughly investigated.

### 5.3 Evaluative cognitions: a proposed ON pathway

Where autistic RRBs may function to support coping with external stimuli, RRBs in ON alternatively function on evaluative internal cognitions. Evaluative cognitions refer to the experience of appraising our own beliefs, behaviours and attributes. As previously outlined, perfectionism may act as the foundation of this development pathway to RRBs. For instance, desire for the perfect diet alongside negative affective responses when not achieved, may result in restriction and repetition as protective methods in order to “achieve perfection” and behave in ways congruent with such evaluative cognitions regarding diet. The evaluative nature of RRBs is also apparent from research addressing attitudes in response to breaking these idealised eating patterns. For example, individuals with ON report, “*I could just have a cup of ice-cream or something, um I feel a little bit bad about that*” and “*There’s more satisfaction when you eat something good just on a more emotional level*” (6). These experiences demonstrate the changing affective responses in relation to whether idealised diet beliefs are obtained, resulting in “satisfaction” compared to when they are contradicted, “*I feel a little bit bad*.” This illustrates RRBs in ON as behavioural strategies to manage discomfort, whereby engaging in restrictive and repetitive eating behaviour keeps such behaviour congruent with the already established perfectionistic ideals. This presents the function of RRBs in ON as a method of reducing self-criticism and managing responsive negative affect, therefore positioning the developmental route to RRBs in ON as self-evaluative.

Although distinguishing self-regulatory and self-evaluative functions may provide a useful conceptual scaffold, these pathways are not mutually exclusive. Whilst self-regulation represents a notable function of autistic RRBs, it is important to acknowledge RRBs, particularly those centred on special interests, may also carry evaluative and identity driven meaning for autistic individuals (57). For ON, engaging in RRBs as a method of ensuring predictability and affective management may represent a similar regulatory-type function displayed in autism (58). Therefore, although a distinction in functional pathways between conditions may be clinically useful, it is necessary to consider both regulatory and evaluative influences, rather than assuming definitive functional pathways in either condition.

The conceptual model presented in **Figure 1** depicts a possible representation of how individual differences, cognitive, affective and functional factors already discussed may work together to produce the outcome of RRBs. In this model blue boxes represent shared components of both autism and ON. Orange boxes represent components more strongly associated with autism, whilst green boxes display components more strongly associated with ON. Green arrows represent pathways supported by strong evidence and red arrows signify pathways supported by preliminary evidence. Although this model highlights anxiety as a key affective factor, other affective factors, such as guilt, pride, and shame, may also contribute to the production and maintenance of RRBs. The model suggests that RRBs function as a behavioural outcome of cognitive and affective factors with the aim of managing sensory information, regulating affective responses, and maintaining congruent self-evaluative beliefs. This, in turn, may increase reliance on RRBs over time in order to continue to cope with uncertainty, cognitive inflexibility and perfectionistic thinking and corresponding anxiety that arises from such cognitions. This model is a tentative illustration of pathways to RRBs across ON and autism, rather than a definitive and exhaustive explanation. Furthermore, the outlined components will likely interact in complex ways, and the weighted influence of each component and pathway will vary between individuals. Although we differentiate pathways with strong and preliminary evidence, further testing of all relationships is necessary to determine which components most reliably predict restrictive behaviour and to clarify the relative contribution of each component in producing the outcome of RRBs.

**Figure 1:**
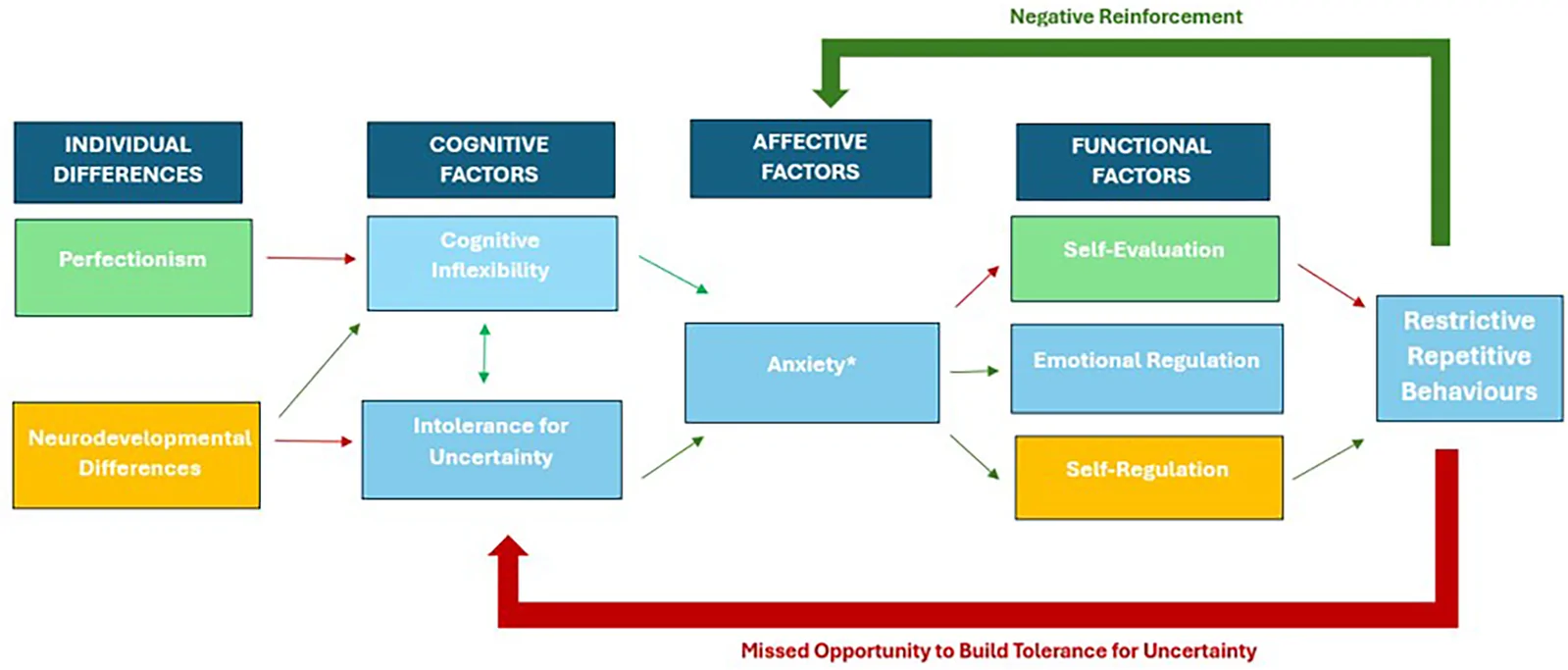
A proposed conceptual model of RRBs across autism and orthorexia nervosa.

## 6 Clinical implications

This perspective highlights the importance of moving beyond surface-level similarities in restrictive repetitive behaviours (RRBs) across autism and orthorexia nervosa (ON) to consider mechanisms. Although behaviours may appear comparable, their underlying mechanisms and functions may differ substantially.

A key clinical priority is comprehensive, individualised assessment and formulation, with a particular focus on identifying the function of RRBs within each presentation. The use of function-based assessment may help determine whether RRBs primarily serve sensory, emotional, or evaluative purposes. In autism, RRBs likely reflect sensory and self-regulatory processes, whereas in ON they may be more closely linked to self-evaluation and perfectionistic beliefs about diet and health. However, across both conditions, RRBs may also function to regulate anxiety and other difficult emotional states, highlighting the relevance of intolerance of uncertainty (IU) and self-regulation as potential transdiagnostic targets for intervention, but this should be carefully adapted to individual needs.

Importantly, the cognitive processes underpinning RRBs differ across conditions, with implications for intervention. In ON, RRBs may be driven by modifiable belief systems (e.g., perfectionism, idealised dietary rules), suggesting the utility of cognitive behavioural approaches. In contrast, in autism, cognitive rigidity may reflect neurodevelopmental differences, and interventions may be more appropriately focused on adaptation and support to be neuro-inclusive. For autistic individuals, RRBs may require sensory-informed accommodations and environmental adjustments, rather than reduction. For anorexia nervosa the PEACE pathway (59) is an effective evidence-based model of care that is neuroaffirmative, considering the needs of autistic individuals. Future work should investigate which elements of the PEACE pathway may be effective for supporting autistic individuals with ON.

Across both populations, clinicians should carefully evaluate whether RRBs are adaptive or maladaptive, recognising that some behaviours may serve important coping functions. Consequently, intervention should be guided by functional impact and individual neuroaffirmative goals, rather than assumptions that all RRBs require modification. Overall, a formulation-driven, person-centred approach is crucial to ensure accurate differentiation and appropriate, effective intervention across ON and autism.

## 7 Conclusion

This perspective offers a conceptual account for how cognitive, affective and functional components may integrate to produce RRBs across autism and ON. Our work highlights the need for clinical services to be aware of the overlap between autism and ON, and for neuroinclusive clinical support for individuals with ON. Our model depicts how RRBs may arise from a combination of shared and distinct mechanisms across conditions. However, empirical testing is required to clarify these proposed pathways. Overall, this conceptualisation highlights the complex nature of RRBs, emphasising a need for function-focused interpretations of complex behavioural presentations. This is necessary to further support clinicians’ in providing appropriate diagnosis, assessment and targeted intervention.

## Data Availability

The original contributions presented in the study are included in the article/supplementary material. Further inquiries can be directed to the corresponding author.
